# Reduction in Hg phytoavailability in soil using Hg‐volatilizing bacteria and biochar and the response of the native bacterial community

**DOI:** 10.1111/1751-7915.13457

**Published:** 2019-06-26

**Authors:** Junjun Chang, Qingchen Yang, Jia Dong, Bohua Ji, Guangzheng Si, Fang He, Benyan Li, Jinquan Chen

**Affiliations:** ^1^ School of Ecology and Environmental Science and Yunnan Key Laboratory for Plateau Mountain Ecology and Restoration of Degraded Environments Yunnan University Kunming 650091 China; ^2^ Institute of International Rivers and Eco‐security Yunnan University Kunming Yunnan 650091 China

## Abstract

Biological approaches are considered promising and eco‐friendly strategies to remediate Hg contamination in soil. This study investigated the potential of two ‘green’ additives, Hg‐volatilizing bacteria (*Pseudomonas* sp. DC‐B1 and *Bacillus* sp. DC‐B2) and sawdust biochar, and their combination to reduce Hg(II) phytoavailability in soil and the effect of the additives on the soil bacterial community. The results showed that the Hg(II) contents in soils and lettuce shoots and roots were all reduced with these additives, achieving more declines of 12.3–27.4%, 24.8–57.8% and 2.0–48.6%, respectively, within 56 days of incubation compared to the control with no additive. The combination of DC‐B2 and 4% biochar performed best in reducing Hg(II) contents in lettuce shoots, achieving a decrease of 57.8% compared with the control. Pyrosequencing analysis showed that the overall bacterial community compositions in the soil samples were similar under different treatments, despite the fact that the relative abundance of dominant genera altered with the additives, suggesting a relatively weak impact of the additives on the soil microbial ecosystem. The low relative abundances of *Pseudomonas* and *Bacillus*, close to the background levels, at the end of the experiment indicated a small biological disturbance of the local microbial niche by the exogenous bacteria.

## Introduction

Soil pollution by heavy metals is a serious environmental problem worldwide (Li *et al*., 2014). Elevated levels of heavy metals in soil pose a severe threat to ecosystem sustainability and human health through the food chain (Li *et al*., 2014; Wang *et al*., 2018). As one of most key global pollutants, mercury (Hg) attracts great concern due to its high mobility and toxicity, increased concentration and long retention in environments (Driscoll *et al*., 2013). Hg has been placed in the third position on the ‘priority list of hazardous substances’ by the Agency for Toxic Substances and Disease Registry (ATSDR, 2014). In addition to the reduction in Hg discharge from anthropogenic activities, development of effective techniques to remediate Hg‐contaminated soil is urgently required for the protection of human and ecosystem health (Wang *et al*., 2012).

Conventional physicochemical approaches such as precipitation and elution with chemical reagents, thermal treatment, solidification and stabilization can effectively remove Hg from soil or transform it to other less toxic or immobile forms (Wang *et al*., 2012; Xu *et al*., 2015). Unfortunately, application of these technologies is usually restricted due to their high cost, secondary pollution and harmfulness to soil ecosystems (Mahbub *et al*., 2017a).

In recent years, the alternative use of eco‐friendly bioagents to remediate Hg‐polluted environments in situ, especially environments with large areas and low Hg concentrations, has attracted increasing interest. Of these bioagents, Hg‐resistant microorganisms and biochar are two emerging and promising representatives (Park *et al*., 2011; Giovanella *et al*., 2017; Mahbub *et al*., 2017a; Chang *et al*., 2019). Specifically, certain microbes can resist Hg pollutants through transforming Hg into volatilizable Hg^0^ with much less toxicity and solubility, which subsequently escapes from Hg‐polluted water or soil employing the ‘*mer*’ operon (Barkay, 1987; Yu *et al*., 2014; Giovanella *et al*., 2016) or immobilizing Hg by various functional groups (Francois *et al*., 2012). To date, many Hg‐resistant bacteria have been isolated; these organisms can remove Hg from solution via volatilization and/or biosorption (Giovanella *et al*., 2016), and a few have recently been used to enhance Hg removal from Hg‐polluted soils (Mahbub *et al*., 2017b; McCarthy *et al*., 2017). Compared to absolute Hg concentration, reducing Hg phytoavailability in soil is more crucial because available Hg can be easily assimilated by plants and may enter the food chain. It is expected that Hg‐volatilizing bacteria can reduce both the Hg content and phytoavailability in soils, but their potential for reducing Hg phytoavailability in soils has not been reported.

Biochar is a carbonaceous material produced by the pyrolysis of carbon‐rich biomass under oxygen‐limited conditions and can remove heavy metals from aqueous solution efficiently through adsorption (Inyang *et al*., 2016). A few recent studies reported that Hg bioavailability in sediment and soil could be decreased by biochar amendment, which is primarily attributed to ion exchange and the complexation of Hg by abundant functional groups such as hydroxy and carbonyl groups on biochar (Liu *et al*., 2016; Wang *et al*., 2018). For soil, other agronomic and environmental advantages such as amelioration of fertility and structure, nutrient fixation, recycling of agricultural waste and carbon sequestration can also be supplied by biochar amendment (Laghari *et al*., 2016; Wang *et al*., 2018). The coapplication of biochar and metal‐resistant bacteria for remediation of Cr‐/Cd‐contaminated soils has been investigated recently (Arshad *et al*., 2017; Li *et al*., 2017, 2018), and our previous work preliminarily showed the feasibility of a Hg‐volatilizing bacterial strain and biochar to enhance Hg elimination from soils (Chen *et al*., 2018). However, little research has focused on the reduction of Hg phytoavailability in soil by biochar and microbes, which is more significant in the remediation of Hg‐polluted soil, and further exploration is needed.

Microbial community structure is critical for the maintenance of the health and stability of soil ecosystems because of the key role of various microorganisms in matter cycling and ecosystem service supply in soil (Wu *et al*., 2016; Fischer *et al*., 2018). Additionally, the microbial community can serve as a bioindicator of environmental variation (Castro *et al*., 2010; Zhang *et al*., 2018). Hg pollution and the introduction of exogenous biomaterials for remediation such as biochar and microorganisms may influence and even disturb the soil microecosystem by changing soil properties and breaking the original microbial niche (Mahbub *et al*., 2017c; Zhang *et al*., 2018). Thus, the function and stability of the soil ecosystem may be adversely impacted, leading to some ecological risk. Unfortunately, relevant information is currently lacking. Therefore, the effect of Hg‐volatilizing bacteria and biochar addition on the microbial community in Hg‐polluted soil must be evaluated.

In this study, two strains of isolated Hg‐volatilizing bacteria and sawdust biochar were added to Hg‐polluted soil for remediation through pot experiments, and the response of the soil bacterial community to these additives was investigated. The main objectives were (i) to investigate the effectiveness of a combined Hg‐volatilizing bacterial and biochar treatment for reducing Hg(II) phytoavailability in Hg‐polluted soil and (ii) to evaluate the effect of these additives on the soil bacterial community.

## Results and discussion

### Effect of Hg‐volatilizing bacteria and biochar additions on Hg contents in soils and lettuces

**Figure 1:**
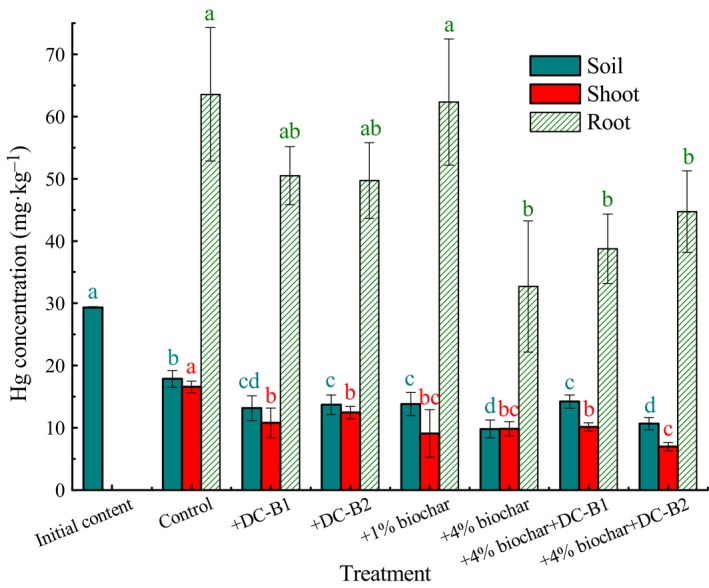
Hg(II) concentrations in soils, lettuce shoots and roots under different treatments. Different letters above the columns indicate significant differences (*P *<* *0.05) between the data in those columns.

The Hg(II) contents in soils and lettuces receiving different treatments are shown in Fig. 1.

The Hg(II) content in the control soil without additives decreased by 39.1%, which might have resulted from abiotic and biotic processes (Schlüter, 2000). Reductants such as Fe^2+^ and dissolved organic compounds may be mainly responsible for the abiotic reduction of Hg(II) (Hori *et al*., 2015), and indigenous microbiota can contribute to Hg elimination (Barkay, 1987). Hg in soil could also be assimilated by the lettuces, but this could be ignored (< 1.0%) because of the small amount of lettuce biomass (Table 1). High Hg losses, accounting for 20–62% of the total initial Hg in soils freshly contaminated with soluble Hg compounds through volatilization during a growing season, were reported in a previous study (Han *et al*., 2006). The amendments of biochar and/or Hg‐volatilizing bacteria DC‐B1 and DC‐B2 promoted Hg removal from the soils by 12.3–27.4% (*P *<* *0.05), with higher efficiency in the microcosms amended with 4% biochar (66.5%) and a combination of 4% biochar and DC‐B2 (63.8%), indicating the ability of these bioagents to reduce Hg content in Hg‐polluted soil in situ. Biochar can act as a reductant by providing electrons and redox‐reactive compounds (e.g. hydroquinone) (Oh *et al*., 2012; Wang *et al*., 2018) and offers habitats for microbes and their interactions, which might greatly facilitate Hg reduction. Chen *et al*. (2016) reported that biochar amendment stimulated bioreduction of Fe(III) and As(V) in sediment. Moreover, soil porosity and aeration can be enhanced by biochar amendment, which facilitates Hg volatilization from soil to air (Laghari *et al*., 2016). Additionally, the Hg volatilization ability of DC‐B1 and DC‐B2 performed to reduce the Hg content in Hg‐polluted soil (55.1% and 53.3%, respectively), although the combination of biochar and Hg‐volatilizing bacteria did not achieve higher Hg removal efficiency as expected, suggesting their potential for augmenting Hg decontamination in soil, which is consistent with recent studies (Mahbub *et al*., 2017b; McCarthy *et al*., 2017).

**Table 1:** Growth and biochemical parameters of lettuces under different treatments

Treatment	Root length (cm plant^−1^)	Shoot length (cm plant^−1^)	Fresh biomass (g plant^−1^)	Protein (μg g^−1^)	Total chlorophyll (mg g^−1^)
Clean	10.73 ± 1.86 a	12.43 ± 0.76 a	2.22 ± 0.12 a	7.11 ± 0.35 b	0.14 ± 0.01 ab
Control	6.6 ± 0.79 c	9.84 ± 1.92 b	1.72 ± 0.32 a	8.11 ± 1.04 b	0.11 ± 0.01 b
+DC‐B1	10.14 ± 2.58 ab	11.66 ± 0.59 a	1.99 ± 0.18 a	7.87 ± 0.85 b	0.14 ± 0.02 a
+DC‐B2	8.47 ± 1.36 ab	10.87 ± 0.76 ab	1.90 ± 0.15 a	7.94 ± 0.41 b	0.12 ± 0.02 bc
+1% biochar	6.80 ± 1.84 c	9.9 ± 0.68 b	1.68 ± 0.06 a	11.43 ± 0.88 a	0.13 ± 0.02 ab
+4% biochar	6.91 ± 0.81 c	10.54 ± 0.06 b	1.8 ± 0.12 a	11.42 ± 1.9 a	0.09 ± 0.00 c
+DC‐B1 + 4% biochar	7.47 ± 1.69 bc	9.57 ± 0.52 b	1.79 ± 0.05 a	9.19 ± 1.72 b	0.10 ± 0.02 c
+DC‐B2 + 4% biochar	6.05 ± 1.78 c	10.47 ± 0.82 b	1.85 ± 0.18 a	8.46 ± 0.55 b	0.09 ± 0.01 c

In addition to the reduction in Hg(II) content in soils, Hg(II) uptake by the lettuce shoots was also significantly decreased (24.8–57.8% relative to the control, *P *<* *0.05) in the biochar and/or Hg‐volatilizing bacteria soil treatments. The lowest Hg(II) uptake by shoots (7.0 ± 0.6 mg kg_dw_^−1^) was observed in the treatment with coapplication of 4% biochar and DC‐B2, although there was no statistically significant difference between this treatment and the treatments with 1% biochar (9.1 ± 3.8 mg kg_dw_^−1^) and 4% biochar (9.8 ± 1.2 mg kg_dw_^−1^). Furthermore, Hg(II) accumulation in lettuce roots also decreased under various treatments (2.0–48.6% relative to the control), although the difference between certain treatments was not significant (control vs. DC‐B1, DC‐B2 and 1% biochar addition, *P *>* *0.05). For comparison, 4% biochar and the combination of 4% biochar and DC‐B2 were better for the remediation of Hg‐polluted soil. In addition to facilitating Hg loss from soils, biochar can bind Hg in a more stable form than unbound Hg by providing abundant functional groups (Inyang *et al*., 2016; Wang *et al*., 2018), lowering the transport and bioavailability of Hg in the soil system. Soil pH values increased from 4.7 to 5.0 and 5.8, respectively, with 1% and 4% biochar addition because of the alkaline nature of the biochar (pH = 9.96), which was conductive to the reduction in Hg bioavailability (Park *et al*., 2011; Arshad *et al*., 2017). DC‐B1 and DC‐B2 transformed Hg(II) to Hg^0^ and might bind a small portion of Hg(II) especially DC‐B1, which is not assimilated by plants (Fernández‐Martínez *et al*., 2015), and consequently, Hg phytoavailability was reduced in soils. Nevertheless, soil is a highly complex ecosystem and the performance of these additives in Hg decontamination in soil can be influenced by many factors. In this study, biochar was more efficient in the remediation of Hg contamination in soil than the Hg‐volatilizing bacteria, and certain treatment such as the combination of 4% biochar and DC‐B1 did not perform well as expected, probably because of the relatively low biomass of bacterial inocula and low Hg volatilization ability and activity of DC‐B1. Further studies on factors influencing the Hg remediation efficiency and corresponding optimization are required. Biochar amendment or bacterial inoculation has reduced plant uptake of heavy metals such as Cd, Cu and Cr (Arshad *et al*., 2017; Li *et al*., 2017, 2018; Liu *et al*., 2018), but to the best of our knowledge, this report is the first concerning the feasibility of remediating Hg‐contaminated soil from the view of Hg volatilization and reduction in Hg bioavailability.

### Effect of biochar and bacterial biomass additions on lettuce growth

The biomass, shoot and root lengths of the lettuce decreased under the stress of Hg and increased in the majority of the microcosms with the additives, although the differences were almost not significant (*P *>* *0.05 except a few treatments, Table 1). Taking the total Hg mass accumulated in lettuce with increased biomass into consideration, the additives also largely reduced Hg uptake by the lettuces. Moreover, protein content in the lettuce shoot from the treatments with biochar (8.46–11.43 μg g^−1^) was higher than that in the control (8.11 μg g^−1^), with significant increase (*P *<* *0.05) in those amended with only biochar, although slight decreases were observed in the bacteria‐added treatments (7.87–7.94 μg g^−1^). Chlorophyll content in the shoot from the microcosms with Hg‐volatilizing bacteria and 1% biochar (0.12–0.14 mg g^−1^) also increased compared with that in the control (0.11 mg g^−1^), while it decreased slightly in other treatments. The lettuce growth parameters were not positively correlated with the Hg remediation efficiency, and lower values were even observed in certain remediated treatments than those in the control, partly as a consequence of the complex influence of the additives on soil properties and growing lettuce. However, the increase of lettuce growth parameters in some treatments indicated that the stress of Hg pollution in soil might have been mitigated by the additives. Biochar can improve soil properties to encourage plant growth, and bacteria belonging to the genera *Pseudomonas* and *Bacillus* may be phosphate‐solubilizing and plant growth‐promoting agents (Canbolat *et al*., 2006; Chen *et al*., 2006). Enhanced plant growth by biochar amendment and/or functional microorganism inoculation in heavy metal‐polluted soil was also recorded in previous reports (e.g. Siripornadulsil and Siripornadulsil, 2013; Rees *et al*., 2016).

### Effect of biochar and DC‐B1/DC‐B2 amendments on the soil bacterial community

A total of 308177 valid bacterial sequences were obtained from the soil samples by high‐throughput sequencing of the 16S rRNA gene and quality control. The shapes of the rarefaction curves (Fig. S1) indicate that bacterial richness was completely sampled. The alpha diversity of the soil bacterial community is shown in Table 2. The bacterial richness was obviously lower in Hg‐polluted soil than in clean soil, suggesting that the number of bacterial populations declined as a result of the Hg stress. The addition of 4% biochar best enhanced bacterial richness, followed by the addition of DC‐B2 and 4% biochar, while 1% biochar amendment could hardly improve bacterial richness, which was consistent with the efficiency of Hg removal from soil by these treatments. The bacterial richness in DC‐B1‐added soils was lower than that in the control, probably because of the antibacterial effect of DC‐B1. Similar bacterial diversity in all soil samples was obtained, indicating that Hg(II) and these additives had little influence on soil bacteria diversity at least in a short incubation time. Similarly, Rutigliano *et al*. (2014) and Papadopoulou *et al*. (2018) reported that biochar addition and bioaugmentation with an isolated thiabendazole‐degrading bacterial consortium showed no effect on the soil bacterial diversity respectively.

**Table 2:** Diversity and richness indices of the bacterial community in the soil samples

Sample	Reads	OTU	Simpson	Shannon	Chao1	ACE
Clean	44 310	2058	0.9832	8.52	2525	2568
Control	37 817	1853	0.9854	8.62	1854	1861
+DC‐B1	33 602	1670	0.9790	8.34	1671	1671
+DC‐B2	37 800	1851	0.9850	8.52	1874	1938
+1% biochar	34 623	1763	0.9842	8.60	1763	1763
+4% biochar	41 949	1951	0.9833	8.51	2176	2278
+DC‐B1 + 4% biochar	38 559	1870	0.9847	8.59	1870	1875
+DC‐B2 + 4% biochar	39 517	1923	0.9851	8.69	1931	1974

**Figure 2:**
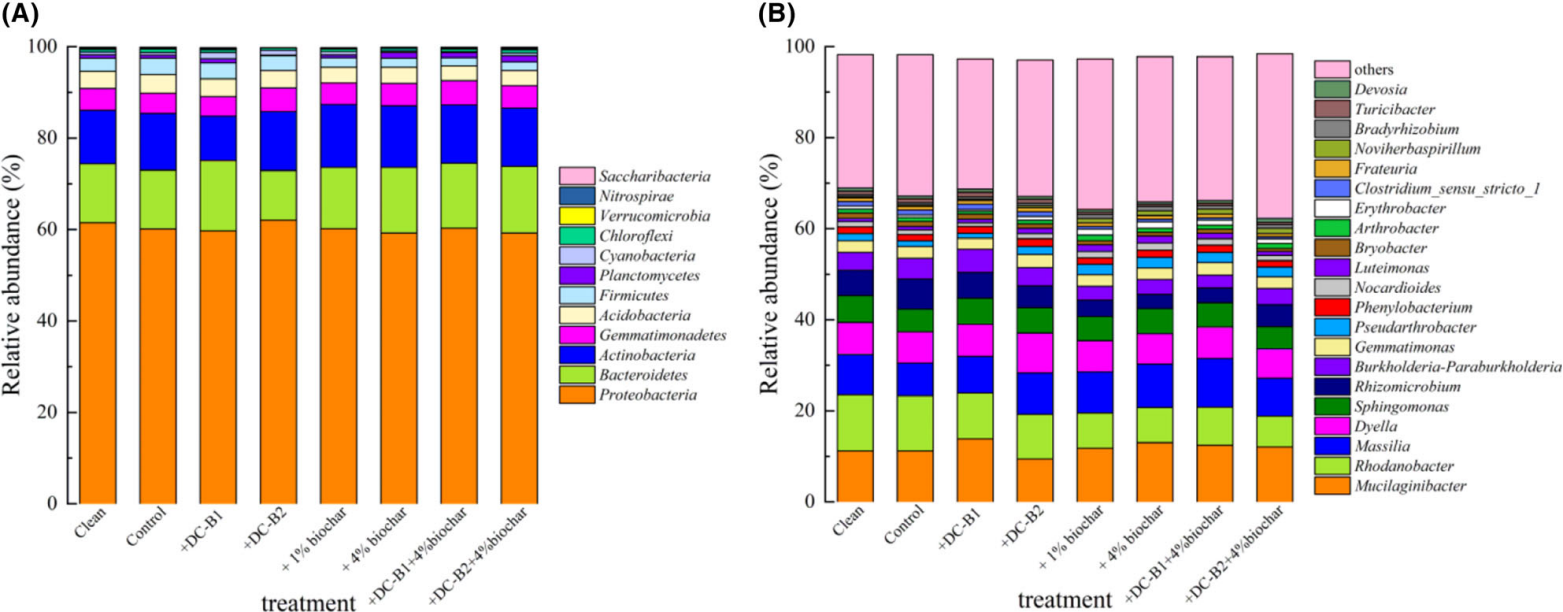
Bacterial community compositions at the phylum (A) and genus (B) levels.

All of the gene sequences were classified from the phylum to the genus level, and the results are shown in Fig. 2. The overall compositions of the bacterial community in the soils under different treatments at the end of the experiment were similar, although the distribution of each phylum varied. *Proteobacteria* occupied the majority (59.2–60.2%) of the total bacterial populations and remained stable under different treatments, suggesting a lack of sensitivity of this phylum to Hg pollution and additives. A previous study also showed that *Proteobacteria* dominated in soil and was less affected by Hg pollution than other phyla (Mahbub *et al*., 2017c). The majority of the spiked Hg was bound with soil particles, and indigenous microbes possessing or developing *mer* operons could reduce part of the available Hg to gaseous Hg^0^ (Barkay, 1987), resulting in a limited influence of Hg on soil bacterial community compositions. Mahbub *et al*. (2017c) observed that bacterial diversity decreased only at a Hg content of more than 50 mg·kg^−1^ in soils. In addition, *Bacteroidetes* (10.8–15.4%), *Actinobacteria* (9.7–13.8%) and *Gemmatimonadetes* (4.4–5.3%) were dominant in the soil samples. The phylum *Actinobacteria* is often associated with the degradation of recalcitrant organic substances, and the increased relative abundance of *Actinobacteria* in the biochar‐amended soil treatments might be derived from their ability to degrade recalcitrant carbon compounds (Lehmann *et al*., 2011). The relative abundances of the copiotrophic phyla *Bacteroidetes* and *Gemmatimonadetes* also increased slightly, and these findings were consistent with previous reports (Xu *et al*., 2016; Sheng and Zhu, 2018). The relative abundance of *Acidobacteria* decreased slightly after biochar addition, probably because the alkaline environment created by biochar amendment is not beneficial for the growth of *Acidobacteria* which are usually acidophilic (Mao *et al*., 2012). Overall, the amendments of these additives did not alter the dominance of the main phyla in the short experimental time, indicating the elasticity of the bacterial community against the disturbance of exogenous introduction of biomaterials in soils.

At the genus level (Fig., 2B), the soil samples showed similar bacterial community compositions, with the dominant genera being *Mucilaginibacter* (9.4–13.8%), *Rhodanobacter* (6.7–12.3%), *Massilia* (7.2–10.7%), *Dyella* (6.4–8.8%), *Sphingomonas* (4.9–5.9%), *Rhizomicrobium* (3.1–6.5%), *Paraburkholderia* (2.8–5.1%), *Gemmatimonas* (2.6–2.8%) and *Pseudarthrobacter* (1.1–2.2%). Although their relative abundances shifted in response to the additives probably because of soil property alteration and competition between exogenous bacteria and native microbiota (Zhang *et al*., 2018), these taxa still dominated under all treatments, suggesting that the additives had a limited impact on the ecological niche of the bacterial community in the soils.

**Figure 3:**
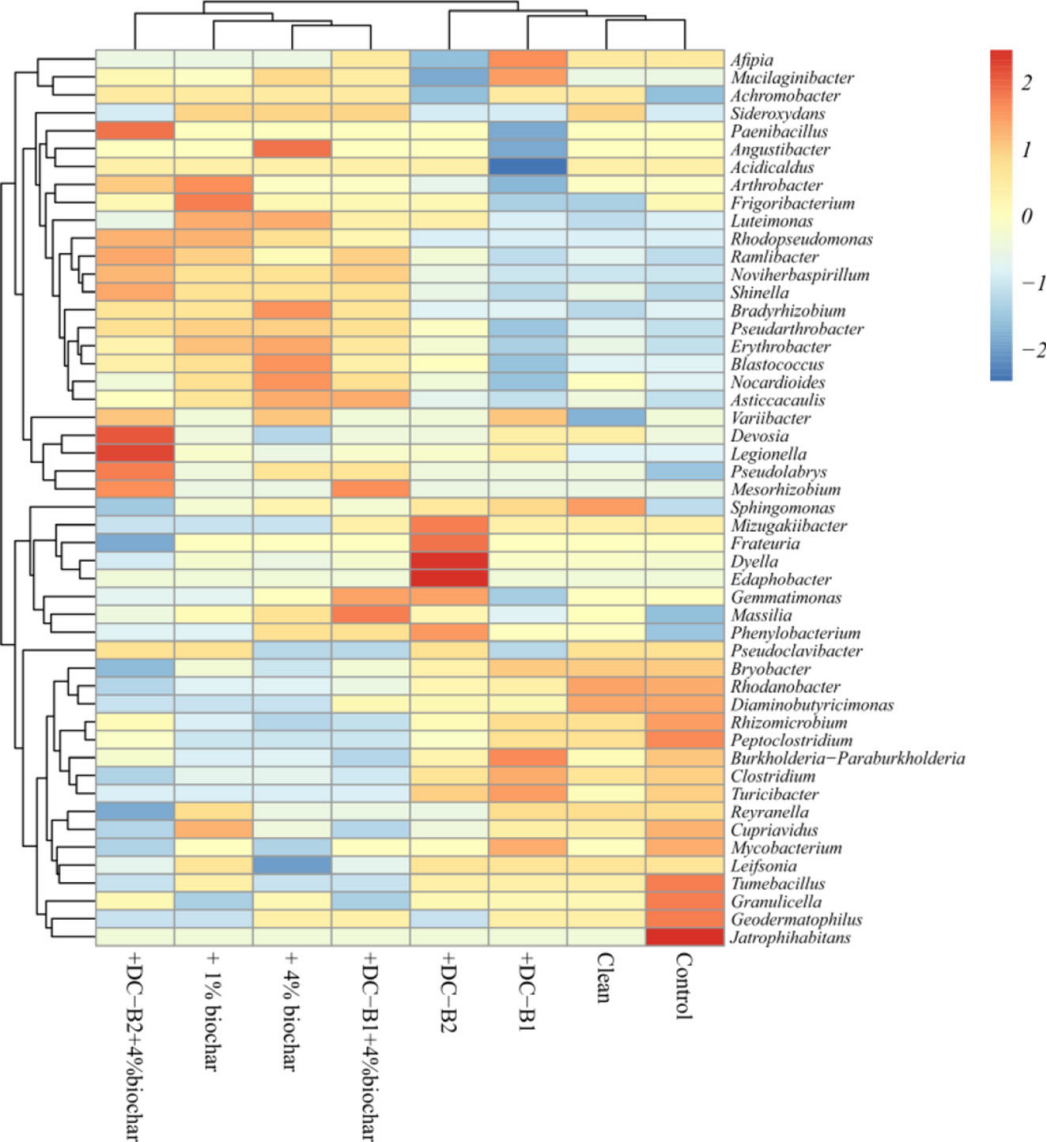
Coloured heat map of the top 50 bacterial genera under different treatments.

The effect of biochar amendment on soil microbial community structure remains controversial. It was reported that wood biochar addition had little or no influence on soil microbial community compositions (Castaldi *et al*., 2011; Liu *et al*., 2017), while in other studies, it was proposed that biochar addition significantly altered microbial community structure and increased microbial activities (Chen *et al*., 2016; Liao *et al*., 2016). This difference may be related to the varied soil and biochar properties and experimental conditions, including incubation time, in these studies (Lehmann *et al*., 2011; Rutigliano *et al*., 2014; Liu *et al*., 2017). In this study with a relatively short operation time, the dominant genera in biochar‐added soils were similar to those in other soils, although their relative abundances shifted and the samples with biochar were clustered together as a group detected by the hierarchically clustered heat‐map analysis (Fig. 3). Specifically, the growth of *Asticcacaulis*,* Noviherbaspirillum*,* Luteimonas*,* Pseudarthrobacter* and *Shinella* was stimulated by biochar amendment (Fig. 3). Among these bacteria, *Pseudarthrobacter* (Zhang *et al*., 2016)*, Shinella* (Bai *et al*., 2009) and *Luteimonas* (Liu *et al*., 2014) may be related to the degradation of recalcitrant aromatic compounds released from the added biochar (Sheng and Zhu, 2018). However, the effect of biochar on microbial populations is relatively limited because microorganisms have difficulty in using most OM from biochar in the short incubation period (Lehmann *et al*., 2011; Wu *et al*., 2016). A similar limited effect of biochar amendment on bacterial community structure in soil remediation has been reported previously (Li *et al*., 2018).

The effect of the invasion of foreign microorganisms on the local microbiota has received increasing attention in recent years (Li *et al*., 2018; Papadopoulou *et al*., 2018). With the soil treatments introducing *Pseudomonas* sp. DC‐B1 and *Bacillus* sp. DC‐B2, the relative abundances of *Bacillus* and *Pseudomonas* were near the low levels (< 0.2%) found in the control (Table S1), indicating that the two added strains vanished at the end of this experiment, which probably indicated the ecological security of the added bacteria with weak influence on the soil microecosystem when applied in soils. A previous study also showed that the number of *Pseudomonas veronii* cells applied directly to Hg‐contaminated soil declined rapidly in the first 3 weeks (McCarthy *et al*., 2017). Similarly, Lang *et al*. (2018) reported that the phylum *Actinobacteria* accounted for the majority of soil bacterial populations after inoculation with the strain AH‐B (belonging to this phylum) for quinclorac degradation, while its relative abundance subsequently decreased to the low level in the control after 56 days of incubation. Homoeostatic mechanisms work to restore the disturbed microbial community to its previous state (Farinati *et al*., 2009). However, further studies should be conducted to maintain the growth and activity of the added bacterial inocula in soils, such as cell immobilization by gel entrapment before addition (Lu *et al*., 2018), to maintain/enhance the Hg remediation efficacy and reduce operation cost.

## Conclusion

This work demonstrated the high potential of two biomaterials—Hg‐volatilizing bacteria DC‐B1/DC‐B2 and biochar—as well as their combinations as additives for the in situ remediation of Hg‐polluted soils by reducing the content and phytoavailability of Hg(II). Moreover, bioaugmentation with these exogenous bioagents displayed a marginal effect on native soil bacterial community structure and diversity, suggesting a weak impact of the additives on the soil microecosystem.

## Experimental procedures

### Materials

#### Hg‐volatilizing bacteria

Two Hg(II)‐volatilizing bacterial strains, *Pseudomonas* sp. DC‐B1 (GenBank No. MG754008, Chen *et al*., 2018) and *Bacillus* sp. DC‐B2 (GenBank No. MG687381, unpublished data), isolated from a heavy metal‐contaminated site previously, were used in this study. These strains displayed a strong ability to remove Hg(II) from culture solution predominantly by volatilization (Hg biosorption by DC‐B1 and DC‐B2 contributed < 15% and 2% for Hg removal, respectively), and the Hg volatilization ability of DC‐B2 was greater than that of DC‐B1. The preliminary study showed that these bacteria had the potential to enhance Hg(II) removal from Hg‐polluted soils.

#### Biochar

Biochar was made by slow pyrolysis of pine sawdust, a by‐product collected from a local wood‐processing factory, and its preparation procedures and basic properties were presented in a previous report (Chen *et al*., 2018). Two application rates of biochar (1% and 4% w·w^−1^ of soil) were used.

### Pot experiments for soil bioremediation

#### Soil sample

The soil sample used for the experiment was collected at depths of 0–5 cm from the Yunnan University campus located in Kunming, China (24°49′12″N, 102°51′4.7″E). After air‐drying and sieving through a 2‐mm sieve, HgCl_2_ solution was dosed into the soils to achieve a Hg content of 40 mg kg^−1^ (dry weight, DW) on the basis of the Hg concentration range in Hg‐contaminated sites recorded in the literature (Qiu *et al*., 2005; Wang *et al*., 2012; Mahbub *et al*., 2017b). After fully homogenizing, the Hg‐spiked soils were stored at room temperature of 20°C for 45 days with the water holding capacity (WHC) maintained at 40–60% for stabilization. Hg(II) content in the soil after ageing was measured to be 29.3 ± 0.13 mg·kg^−1^, suggesting that a fraction of the Hg had been volatilized.

Basic physicochemical properties of the soil sample were determined based on the methods described previously (Yang *et al*., 2008; Chen *et al*., 2018) and listed as follows: pH 4.71 ± 0.08, organic matter content 0.23 ± 0.03%, available phosphate 1.21 ± 0.06 mg·kg^−1^, potassium 123.34 ± 6.81 mg·kg^−1^, alkaline nitrogen 83.7 ± 7.12 mg·kg^−1^and sandy loam texture.

#### Experimental setup

The bioremediation experiment was performed in a greenhouse at Yunnan University from October to December 2017. The Hg‐containing soils (200 g) were put in polypropylene pots (length × width × height: 6 × 7.5 × 8.3 cm), and DC‐B1 and DC‐B2 inocula developed in LB medium overnight to early log phase were added to reduce Hg content and phytoavailability in soil at a dosage of 10^7 ^cfu g^−1^ soil (Arshad *et al*., 2017). In addition, two dosages of biochar, that is, 1% and 4% (w/w), were fully incorporated into the soils. Sterile distilled water was supplied regularly to maintain a WHC of approximately 50% throughout the experiment. In total, eight experimental groups (three replicates per group) were set up: (i) Original soils (Clean); (ii) Hg‐polluted soils with no additive (Control); (iii) Hg‐polluted soils with DC‐B1 (+DC‐B1); (iv) Hg‐polluted soils with DC‐B2 (+DC‐B2); (v) Hg‐polluted soils amended with 1% biochar (+1% biochar); (vi) Hg‐polluted soils with 4% biochar (+4% biochar); (vii) Hg‐polluted soils amended with DC‐B1 and 4% biochar (+DC‐B1 + 4% biochar); and (viii) Hg‐polluted soils with DC‐B2 and 4% biochar (+DC‐B2 + 4% biochar). After seven days of pretreatment, lettuce seeds were well distributed into the soils at a density of eight seeds/pot to determine the germination rate. The lettuce plants were thinned to three seedlings per pot after germination (7 days) to facilitate their growth and further experimental observation. Lettuce is a popular species of vegetable worldwide and can also be employed as an effective bio‐indicator of Hg contamination in soil (Mahbub *et al*., 2017b). Then, soil and plant samples were harvested for analysis after 56 days of cultivation. A portion of soil samples were air‐dried and grounded, and the others were freeze‐dried and stored at −20°C for microbiological analysis.

#### Sample analysis

Lettuce plants were separated into above‐ground (shoot) and below‐ground (root) parts after cleaning with sterile water, and their growth parameters, including lengths of shoots and roots, fresh weight and contents of protein and chlorophyll in shoots, were measured. The protein content was determined using a commercial protein kit (Jiancheng Bioengineering Institute, China), and chlorophyll was extracted using 80% (w/w) acetone and measured by spectrophotometry (Wang *et al*., 2016). Then, the plant was oven‐dried at 50°C to a constant weight. Soil and plant samples were digested following the methods described by Zheng *et al*. (2008) and Han *et al*. (2006) respectively. The Hg concentration in the solutions was determined using a hydride generation atomic fluorescence spectrometer (AFS‐8220; Jitian Analytical Instrument Co., Beijing, China) (Chen *et al*., 2018). Certified reference soil (GBW‐07405, 0.29 ± 0.03 mg Hg kg^−1^) and tomato leaves (CRM 1753a, 34 ng Hg g^−1^) were also determined following the same procedures, and the recovery percentages of Hg were 90–98%.

### Responses of the soil bacterial community

#### DNA extraction

Extraction of soil DNA was performed using a PowerSoil^®^ DNA Isolation Kit (MoBio Laboratories, Carlsbad, CA, USA) according to the manufacturer's protocols. The extracted DNA was purified utilizing the TIANquick Midi Purification Kit (TIANGEN, Beijing, China), and its purity was checked by electrophoresis. Then, the product was stored at −20°C until further analysis. Triplicate purified DNA samples from each group were mixed and sequenced as one sample.

#### Bacterial community analysis

The V3‐V4 region of the bacterial 16S rRNA gene was amplified with universal primers 338F (5′‐ACTCCTACGGGAGGCAGCA‐3′) and 806R (5′‐GGACTACHVGGGTWTCTAAT‐3′) (Xu *et al*., 2016). The purified PCR products were analysed using commercial Illumina MiSeq sequencing technology (Person Biotechnology Co., Ltd, Shanghai, China) to obtain bacterial community information following the methods and program described in previous reports (Chen *et al*., 2016; Xu *et al*., 2016). The obtained raw reads were processed using the QIIME Pipeline (Version 1.8.0, http://qiime.org/). After filtering low‐quality or ambiguous reads and removing chimeric sequences, the high‐quality sequences were clustered into operational taxonomic units (OTUs) at an identity level of 97%, and bacterial taxonomy was assigned using the Ribosomal Database Project (RDP) classifier. Shannon and Simpson indices were calculated to indicate bacterial diversity, and the abundance‐based coverage estimator (ACE) and the estimated asymptotic microbial taxon richness (Chao1) were used to evaluate the bacterial richness. The Bray–Curtis distance was calculated using the dominant genera, and a heat map depicting the changes in bacterial communities was drawn using r software.

### Statistical analysis

All of the assays in this study were performed in triplicate, and the results were expressed as mean value ± SD. Treatment effects on Hg contents in soils and plants were analysed by one‐way analyses of variance (anova) followed by the least significant difference (LSD) using spss 23.0, and a difference was considered significant at the *P *<* *0.05 level. All data were tested for normality and homogeneity of variance before anova. Of these data, lettuce shoot length and total biomass were not normally distributed, and Dunnett's T3 *post hoc* test was applied for analysis.

## Conflict of interest

None declared.

## Supporting information


**Fig. S1**. Rarefaction curves of bacterial sequences in soil samples under different treatments.
**Table S1.** Relative abundance (%) of some representative bacteria in the genus level in different soil treatments.
