# Supplementary material for: Reduction in Hg phytoavailability in soil using Hg‐volatilizing bacteria and biochar and the response of the native bacterial community
Source: Microb Biotechnol. 2019 Jun 26;12(5):1014–23. doi: 10.1111/1751-7915.13457 (PMC6681405; doi:10.1111/1751-7915.13457)
Supplement: Supplementary file 1 — Fig. S1. Rarefaction curves of bacterial sequences in soil samples under different treatments. Table S1. Relative abundance (%) of some representative bacteria in the genus level in different soil treatments. [file MBT2-12-1014-s001.doc]

Fig. S1 Rarefaction curves of bacterial sequences in soil samples under different treatments

Table S1 Relative abundance (%) of some representative bacteria in the genus level in different soil treatments

| Genus | Clean | Control | +DC-B1 | +DC-B2 | +1% biochar | +4% biochar | +DC-B1+4% biochar | +DC-B2+4% biochar |
| --- | --- | --- | --- | --- | --- | --- | --- | --- |
| *Pseudarthrobacter* | 1.5 | 1.3 | 1.1 | 1.8 | 2.3 | 2.3 | 2.2 | 2.2 |
| *Luteimonas* | 0.7 | 0.8 | 0.8 | 1.2 | 1.5 | 1.5 | 1.2 | 0.9 |
| *Shinella* | 0.2 | 0.1 | 0.1 | 0.2 | 0.4 | 0.4 | 0.4 | 0.5 |
| *Rhodopseudomonas* | 0.2 | 0.2 | 0.2 | 0.2 | 0.6 | 0.5 | 0.4 | 0.6 |
| *Noviherbaspirillum* | 0.3 | 0.3 | 0.3 | 0.5 | 1.0 | 1.0 | 1.1 | 1.2 |
| *Asticcacaulis* | 0.4 | 0.1 | 0.1 | 0.3 | 0.9 | 1.2 | 1.2 | 0.6 |
| *Ramlibacter* | 0.3 | 0.2 | 0.2 | 0.4 | 0.7 | 0.5 | 0.7 | 0.8 |
| *Pseudolabrys* | 0.4 | 0.3 | 0.4 | 0.4 | 0.4 | 0.5 | 0.5 | 0.6 |
| *Blastococcus* | 0.4 | 0.4 | 0.2 | 0.6 | 0.8 | 1.0 | 0.7 | 0.7 |
| *Nitrospira* | 0.1 | 0.2 | 0.2 | 0.1 | 0.1 | 0.1 | 0.1 | 0.2 |
| *Nitrosospira* | 0.1 | 0.1 | 0.1 | 0.1 | 0.1 | 0.1 | 0.1 | 0.1 |
| *Nitrobacter* | 0 | 0 | 0 | 0 | 0.1 | 0.1 | 0.1 | 0.1 |
| *Pseudomonas* | 0.1 | 0.2 | 0 | 0 | 0 | 0 | 0 | 0 |
| *Bacillus* | 0.1 | 0.1 | 0.1 | 0.1 | 0.1 | 0 | 0.1 | 0 |
